# Letter to the Editor: Use of Polydeoxyribonucleotide, Retinoic Acid, and Laser in the Treatment of Postinflammatory Hyperpigmentation

**DOI:** 10.1111/jocd.70433

**Published:** 2025-08-31

**Authors:** Rafael Rodrigo Crisanto de Oliveira

**Affiliations:** ^1^ Midas Clinic Ipueiras Brazil

To the Editor,

Regenerative medicine is a promising area that offers safe and effective treatments for several conditions. Among these, postprocedure complications may occur; hence, the need for an adequate preprocedure approach that provides safety to the patient.

The objective of this study was to describe the protocol used for facial whitening after postprocedure complications with trichloroacetic acid and postinflammatory hyperpigmentation (PIH), making it important to publicize it, given the satisfactory result.

On September 20, 2024, a peeling with 20% trichloroacetic acid was performed on the patient, who was advised not to expose herself to the sun. Upon returning on October 28, the patient presented intense PIH in the mandibular, zygomatic, malar, nasal, frontal, orbicularis oris, and eye regions after sun exposure, as shown in Figure 1.

**FIGURE 1:**
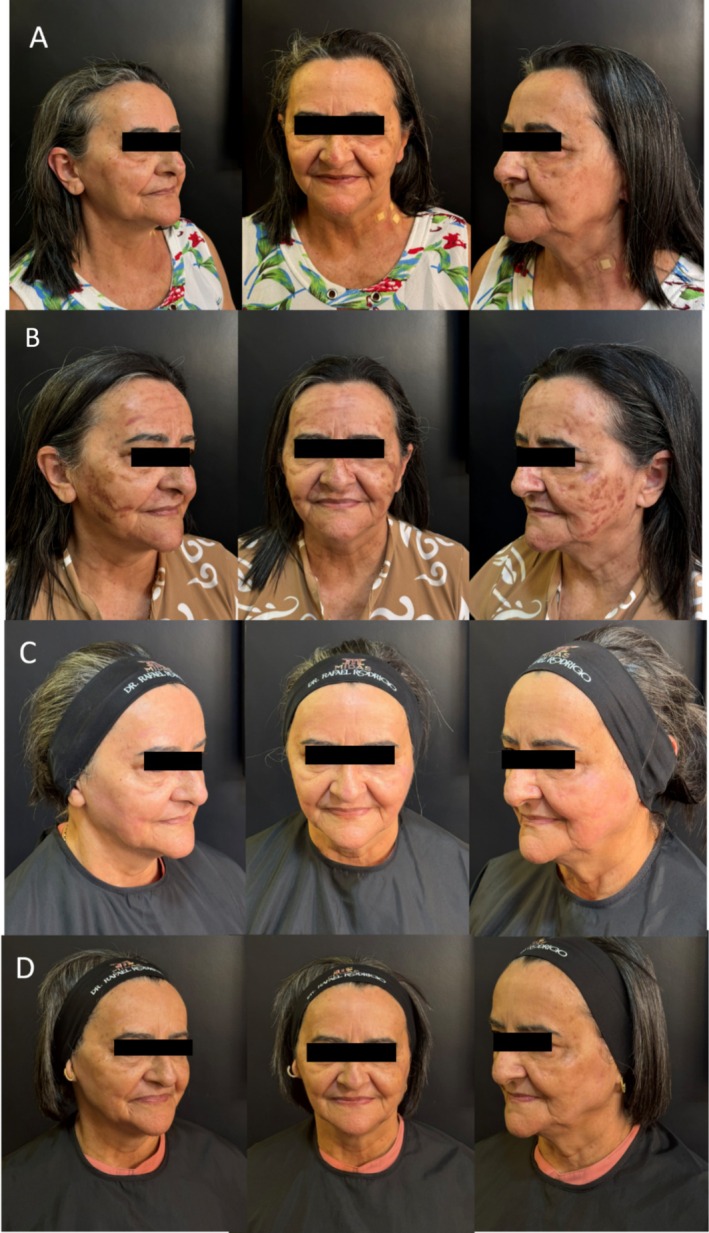
(A) Patient before the procedure on September 02, 2024. (B) HPI on October 28, 2024. (C) Assessment on January 07, 2025, after the protocol indicated for the treatment. (D) Follow‐up comparing the effectiveness of the protocol used in the treatment on April 30, 2025. *Source:* Record made by the author.

Polydeoxyribonucleotide (PDRN) is composed of deoxyribonucleotides with various effects, including tissue repair, anti‐ischemic, anti‐inflammatory, and whitening properties. Additionally, it reduces metalloproteinases and proteoglycan degradation, stimulates osteoblasts, and works synergistically with glucosamine, thereby improving extracellular matrix integrity [1, 2]. Derived from salmon sperm, it undergoes purification to eliminate peptides and proteins that may trigger immune reactions. In vivo studies indicate that PDRN enhances A2a receptor activity and supplies nucleotides and nucleosides to cells, promoting cell regeneration and DNA formation, as shown in vitro. It has been effective in accelerating skin repair in diabetic models and has demonstrated double the success in wound healing compared to placebo in human studies [3, 4].

The Q‐switched ND:YAG 1064 nm laser emits energy in short pulses, effectively targeting melanocytes in the epidermis and dermis to break down melanin particles for lymphatic absorption. This laser is widely utilized for lightening pigmented lesions, such as Becker's nevus, melasma, café‐au‐lait spots, and Argyria [5, 6]. Its inclusion in this treatment protocol was essential due to the facial darkening observed after the peeling procedure.

In view of this, a strategic plan was drawn up to recover this skin and lighten the PIH, including a product composed of 44 mg of hyaluronic acid, 2.2 mg of niacinamide, and 11 mg of polydeoxyribonucleotide (PDRN). This was followed by the use of PDRN, retinoic acid, ND:YAG 1064 nm laser, and topical home medications to improve the condition. To apply the planned procedure protocol, the regions were analyzed, starting with PDRN and retinoic acid. With significant signs of skin lightening; however, with visible PIH on November 14, a session of ND:YAG 1064 nm laser was performed in addition to 10% retinoic acid, being prescribed for home use a formula with 4% hydroquinone, 0.05% tretinoin, and 0.01% fluocinolone acetonide. The patient had no reported comorbidities.

When returning on December 14, the patient presented better lightening of the PIH, more regular skin, with significant satisfaction, but still presented hyperchromic plaques in a lighter tone. Another session of ND:YAG 1064 laser and intradermal injection of PDRN with niacinamide and hyaluronic acid was performed.

On January 7, 2025, the patient returned with substantial and visible improvement of the PIH, erythematous skin coloration in areas of previous PIH, instead of hyperpigmentation. On April 30, a new evaluation comparing the effectiveness of the protocol used is seen in Figure 1.

Injectable PDRN was chosen in this specific case, although it is still questionable worldwide, since the patient had suffered an unintentional removal of part of the epidermis and her treatment aimed to recover fragile, sensitized skin with significant thinning secondary to the previous procedure. Based on clinical evaluation, it was decided to perform the procedure through intradermotherapy, by injection instead of ablative post‐laser or microneedling, even though this was the first choice, since it would remove the protective layer of the skin, which was precisely the intention of her treatment plan.

During the treatment, using retinoic acid, the patient presented flaking, erythema, and sensitivity to heat, which were expected and duly advised. There were no complaints during PDRN and laser applications.

Based on the planning outlined for the patient in question, it is not possible to determine which product was the determining factor in obtaining the satisfactory result or whether it was the combination of the procedures performed, but achieving the objective is what really matters to support future treatments.

Considering the complications, we need to have tools available to provide complete care to the patient, safely, in unplanned situations. Thus, it is possible to exemplify the importance of combined regenerative therapy containing PDRN, niacinamide, and hyaluronic acid, associated with home whitening products, in‐office retinoic acid, and 1064 nm ND:YAG laser in the recovery process in case of post‐inflammatory hyperpigmentation secondary to unexpected PIH after peeling with trichloroacetic acid, bringing a regenerative and whitening response to the patient, in addition to indicating the safety of the application of this product intradermally. For more evidence, other studies are necessary.

## Conflicts of Interest

The author declares no conflicts of interest.

## Supporting information


**Data S1:** jocd70433‐sup‐0001‐Supinfo01.zip.

## Data Availability

The data that support the findings of this study are openly available in Refs. [1, 2, 3, 4, 5, 6].
