# Supplementary figures and images for: Letter to the Editor: Use of Polydeoxyribonucleotide, Retinoic Acid, and Laser in the Treatment of Postinflammatory Hyperpigmentation
Source: J Cosmet Dermatol. 2025 Aug 31;24(9):e70433. doi: 10.1111/jocd.70433 (PMC12399916; doi:10.1111/jocd.70433)

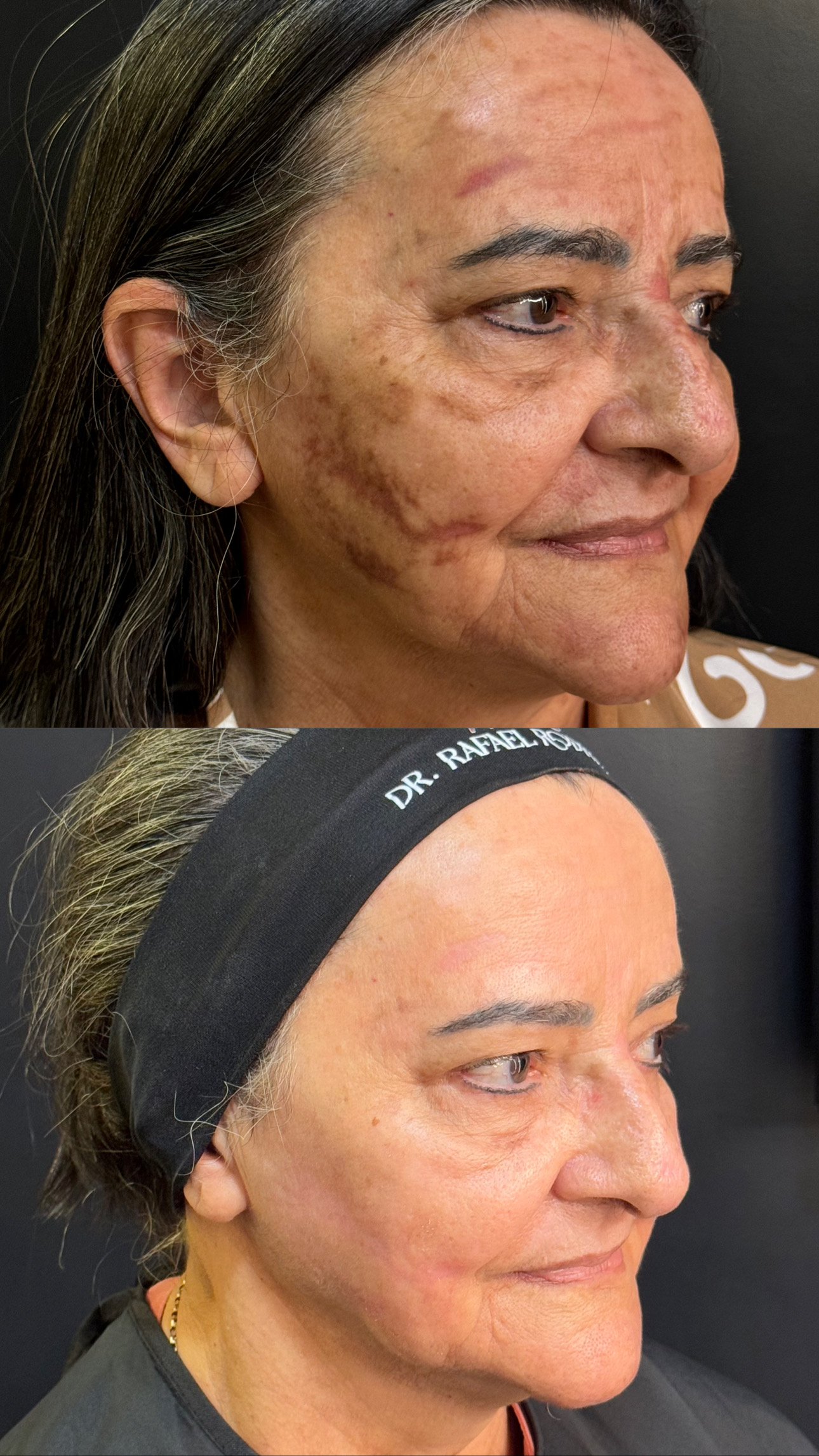

Supplement: Supplementary file 1 — Data S1: jocd70433‐sup‐0001‐Supinfo01.zip. [file JOCD-24-e70433-s001.zip › JOCD_70433_f15_15BC47E4-41F0-4EBE-BC5E-918A88D0BDF0.jpg]

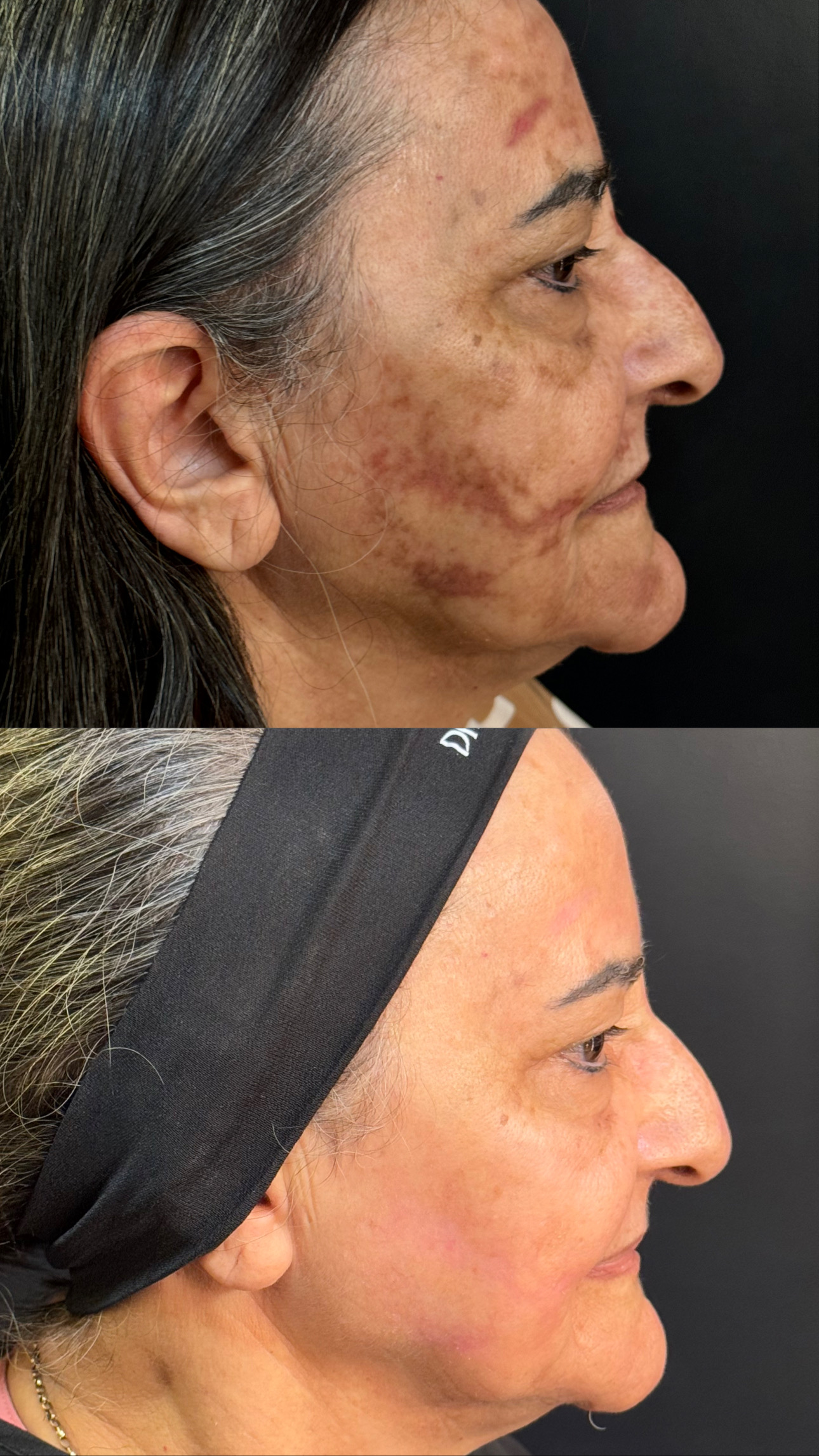

Supplement: Supplementary file 1 — Data S1: jocd70433‐sup‐0001‐Supinfo01.zip. [file JOCD-24-e70433-s001.zip › JOCD_70433_f39_A39B203D-801C-45EE-919F-FE42C5E54582.jpg]

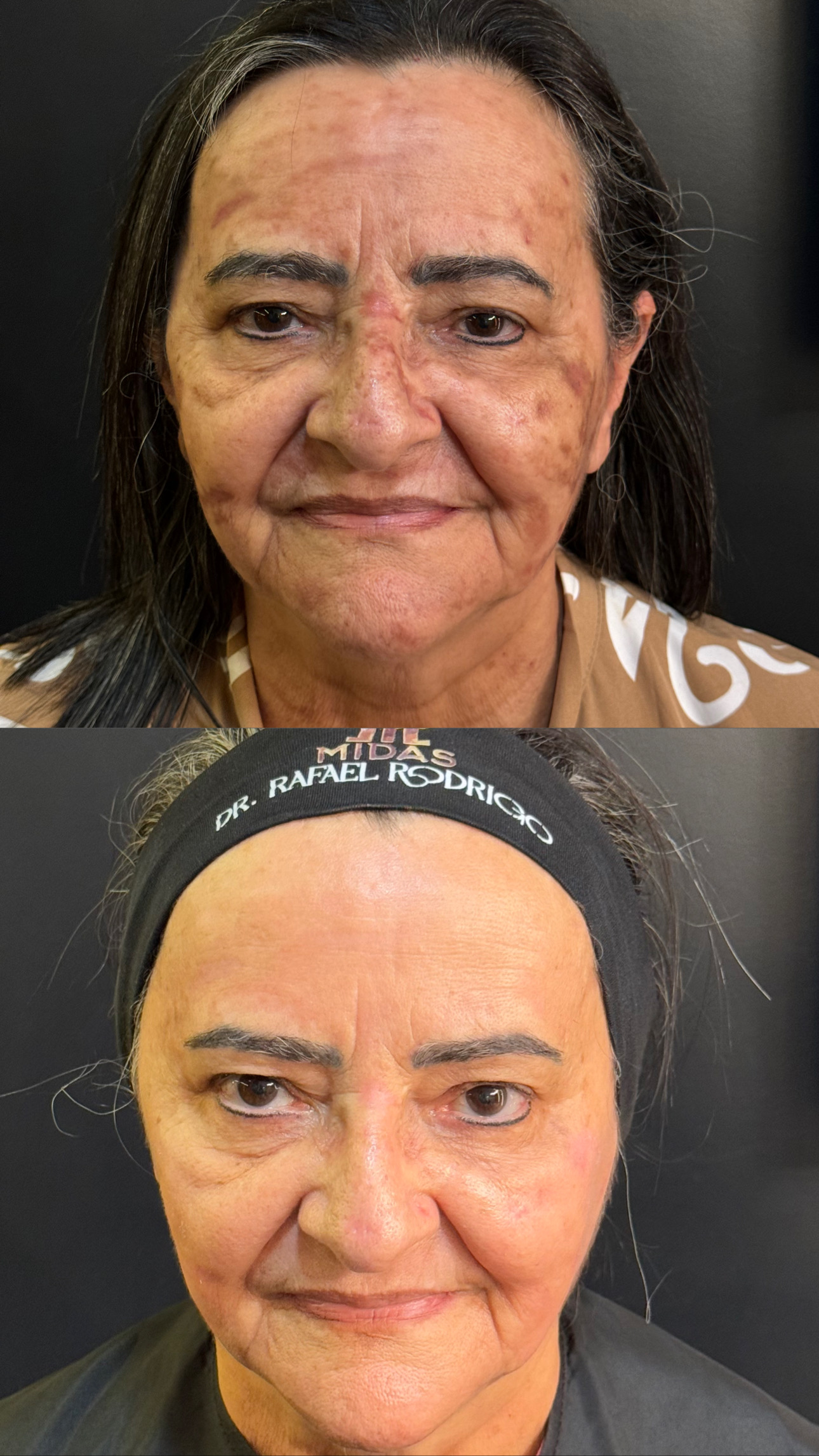

Supplement: Supplementary file 1 — Data S1: jocd70433‐sup‐0001‐Supinfo01.zip. [file JOCD-24-e70433-s001.zip › JOCD_70433_f62_62C62DA0-1389-4E6C-A42D-AF630F6202D0.jpg]

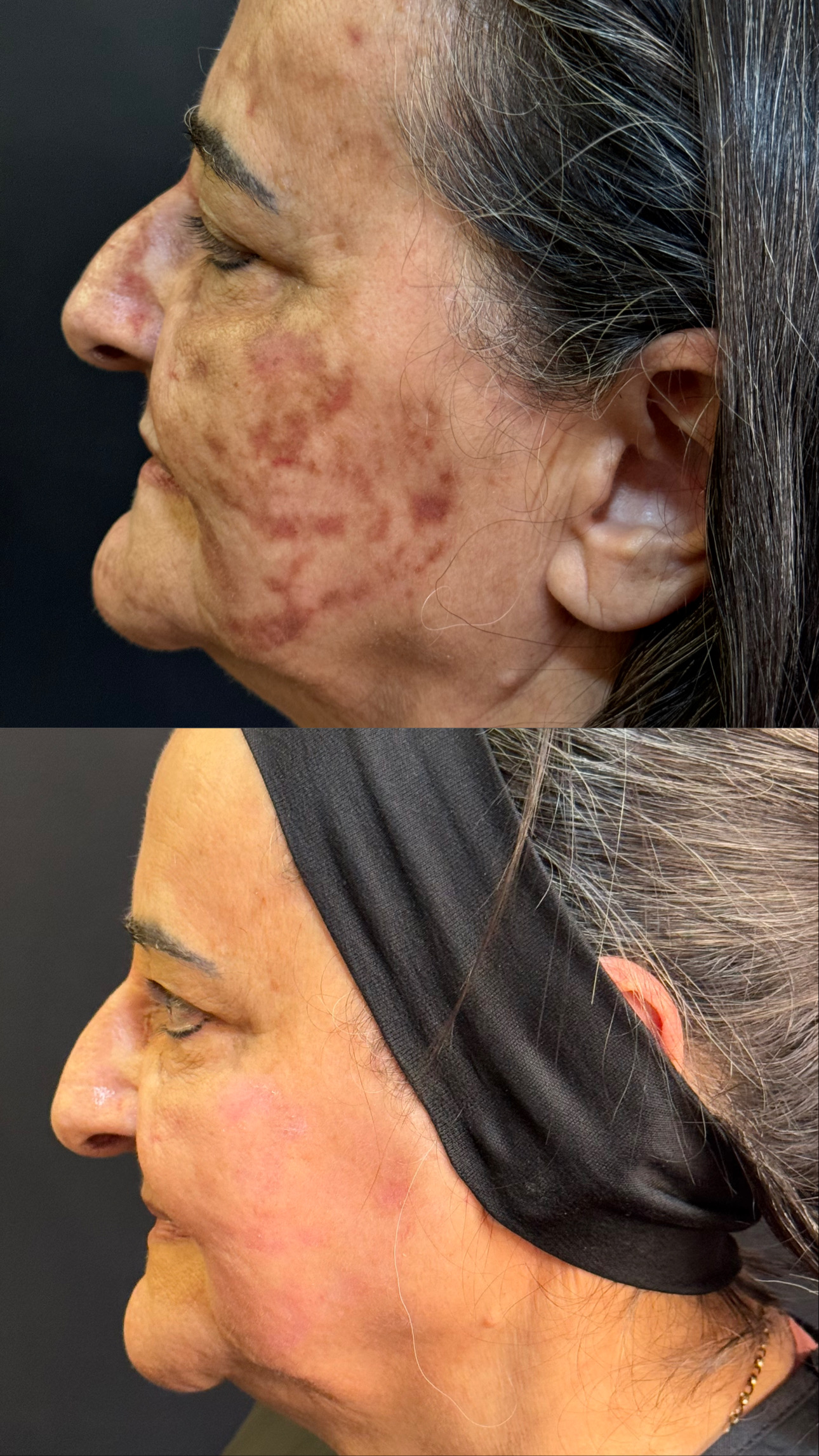

Supplement: Supplementary file 1 — Data S1: jocd70433‐sup‐0001‐Supinfo01.zip. [file JOCD-24-e70433-s001.zip › JOCD_70433_f91_AC91B713-4EA5-47DE-ABAF-B589A6B41D56.jpg]

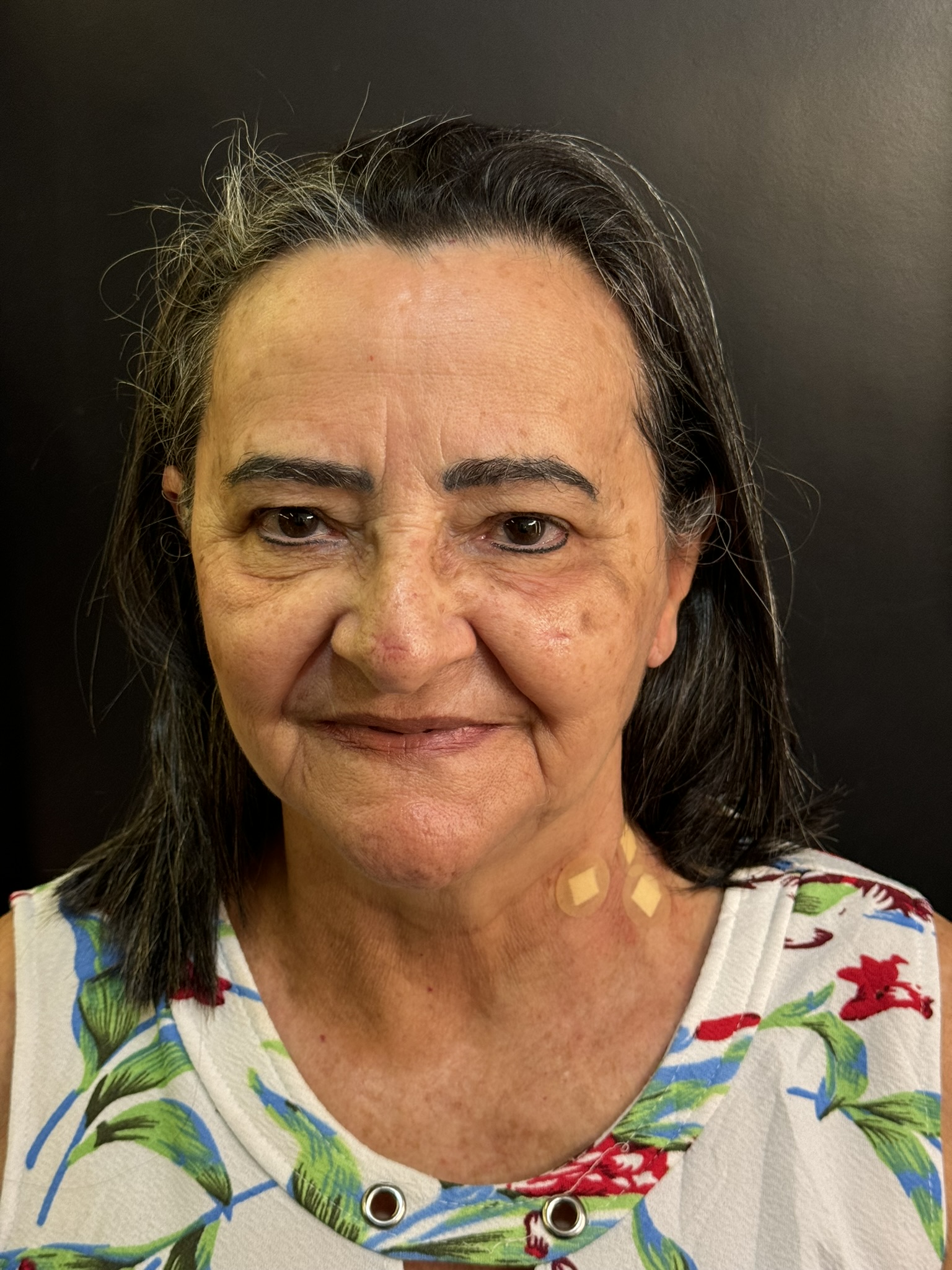

Supplement: Supplementary file 1 — Data S1: jocd70433‐sup‐0001‐Supinfo01.zip. [file JOCD-24-e70433-s001.zip › before.JPEG]

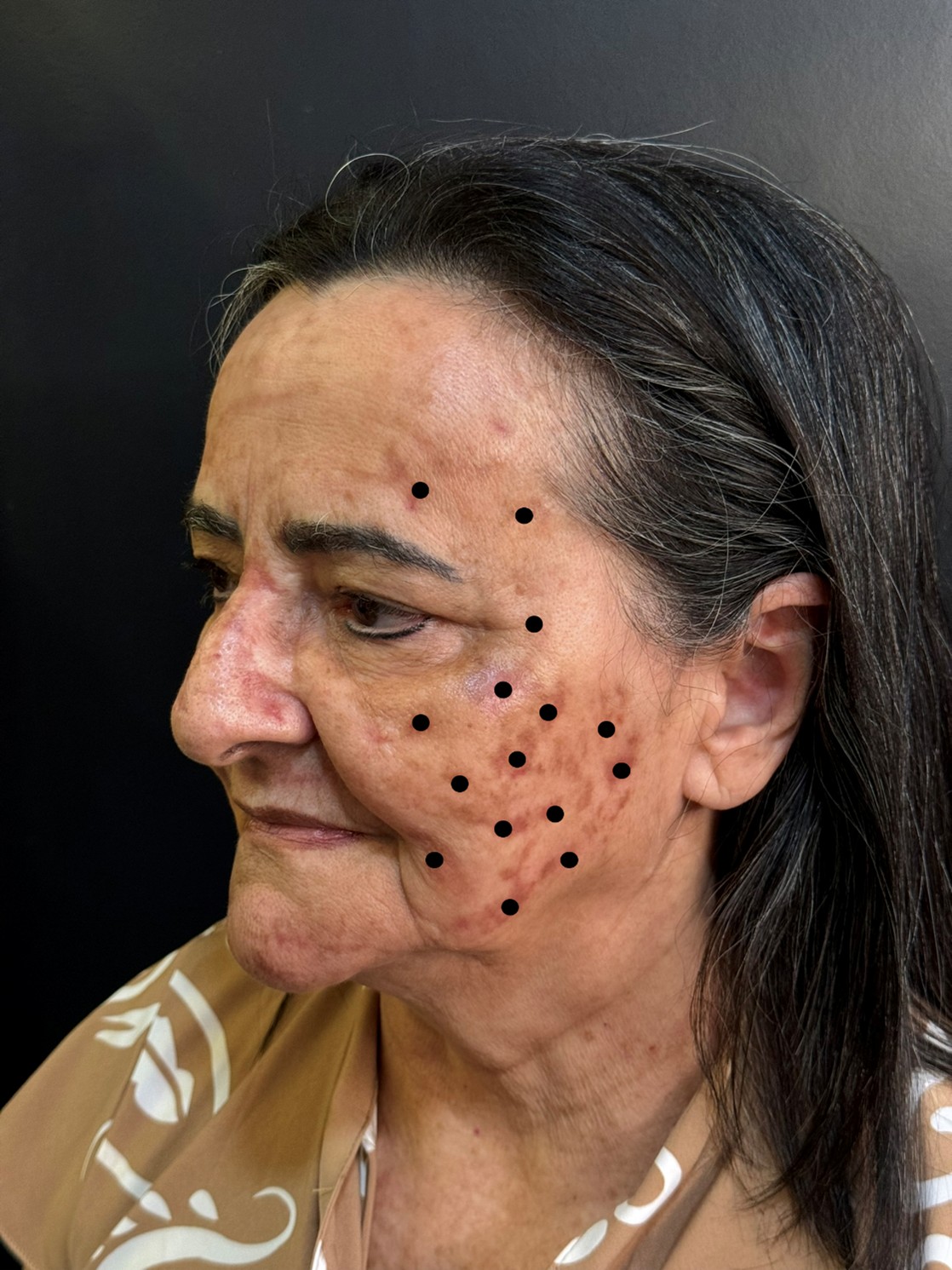

Supplement: Supplementary file 1 — Data S1: jocd70433‐sup‐0001‐Supinfo01.zip. [file JOCD-24-e70433-s001.zip › Imagem1.jpg]

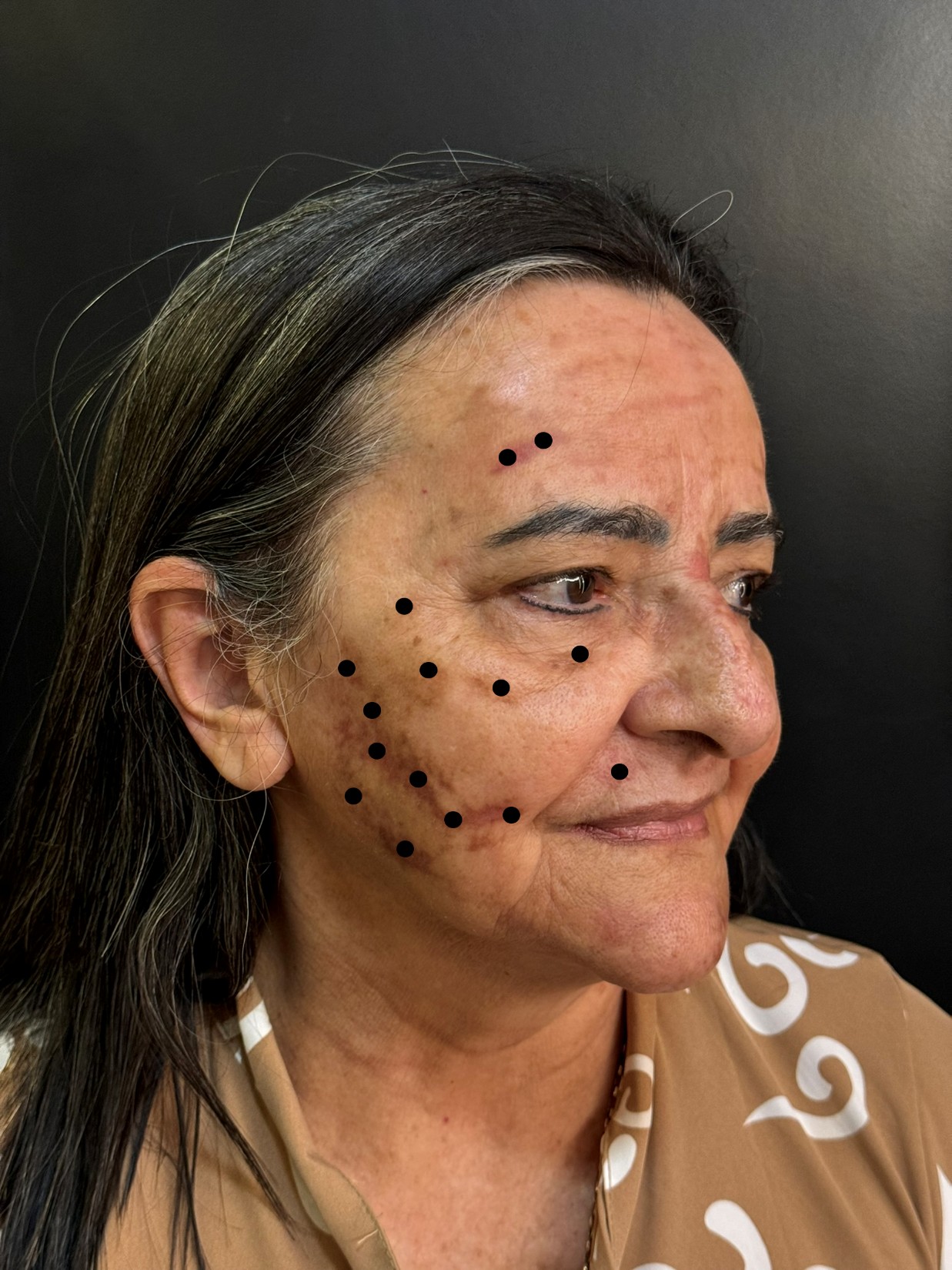

Supplement: Supplementary file 1 — Data S1: jocd70433‐sup‐0001‐Supinfo01.zip. [file JOCD-24-e70433-s001.zip › Imagem2.jpg]

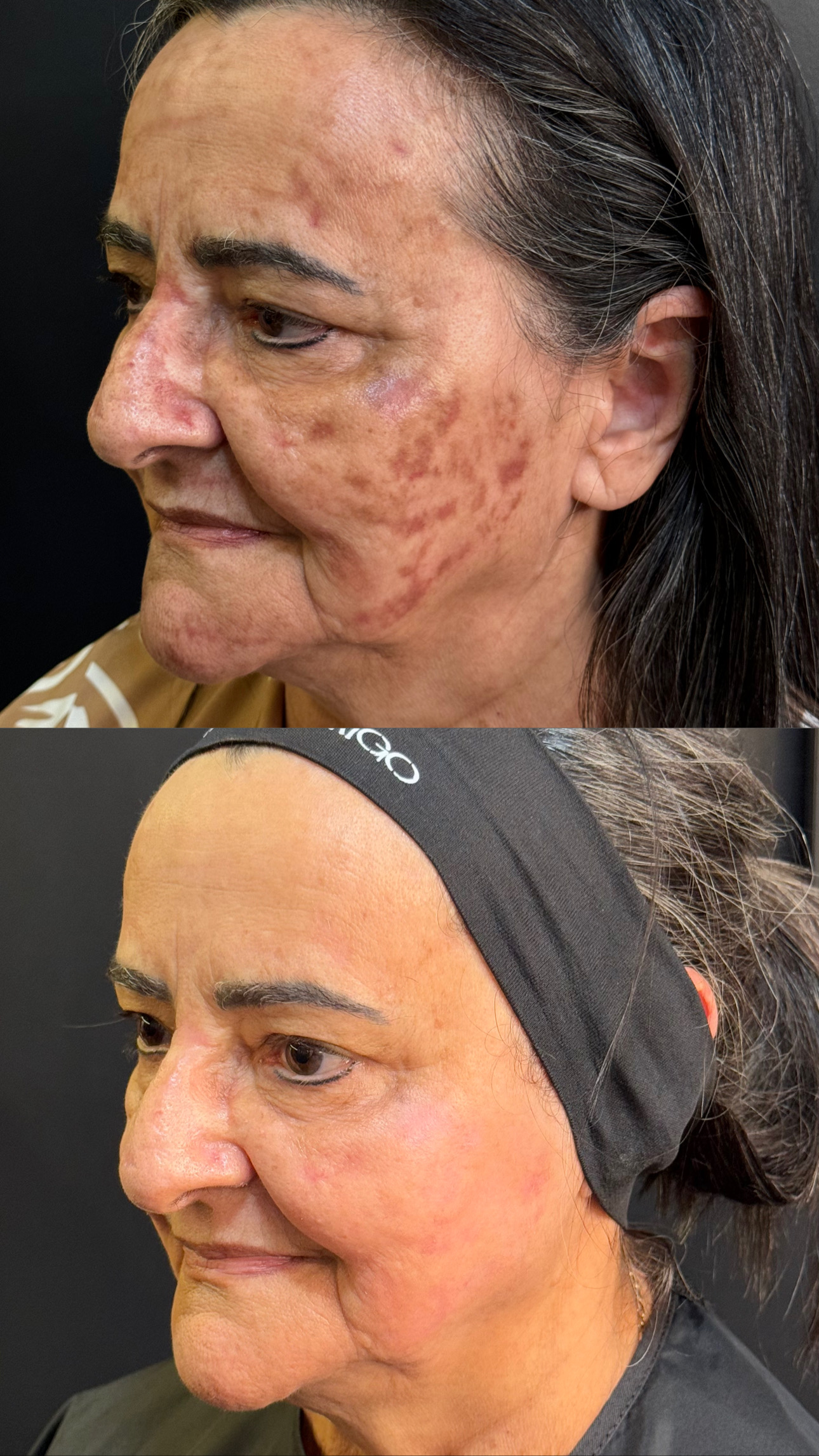

Supplement: Supplementary file 1 — Data S1: jocd70433‐sup‐0001‐Supinfo01.zip. [file JOCD-24-e70433-s001.zip › JOCD_70433_f1_1D7CC625-841C-4639-9F26-FB9297B507E4.jpg]

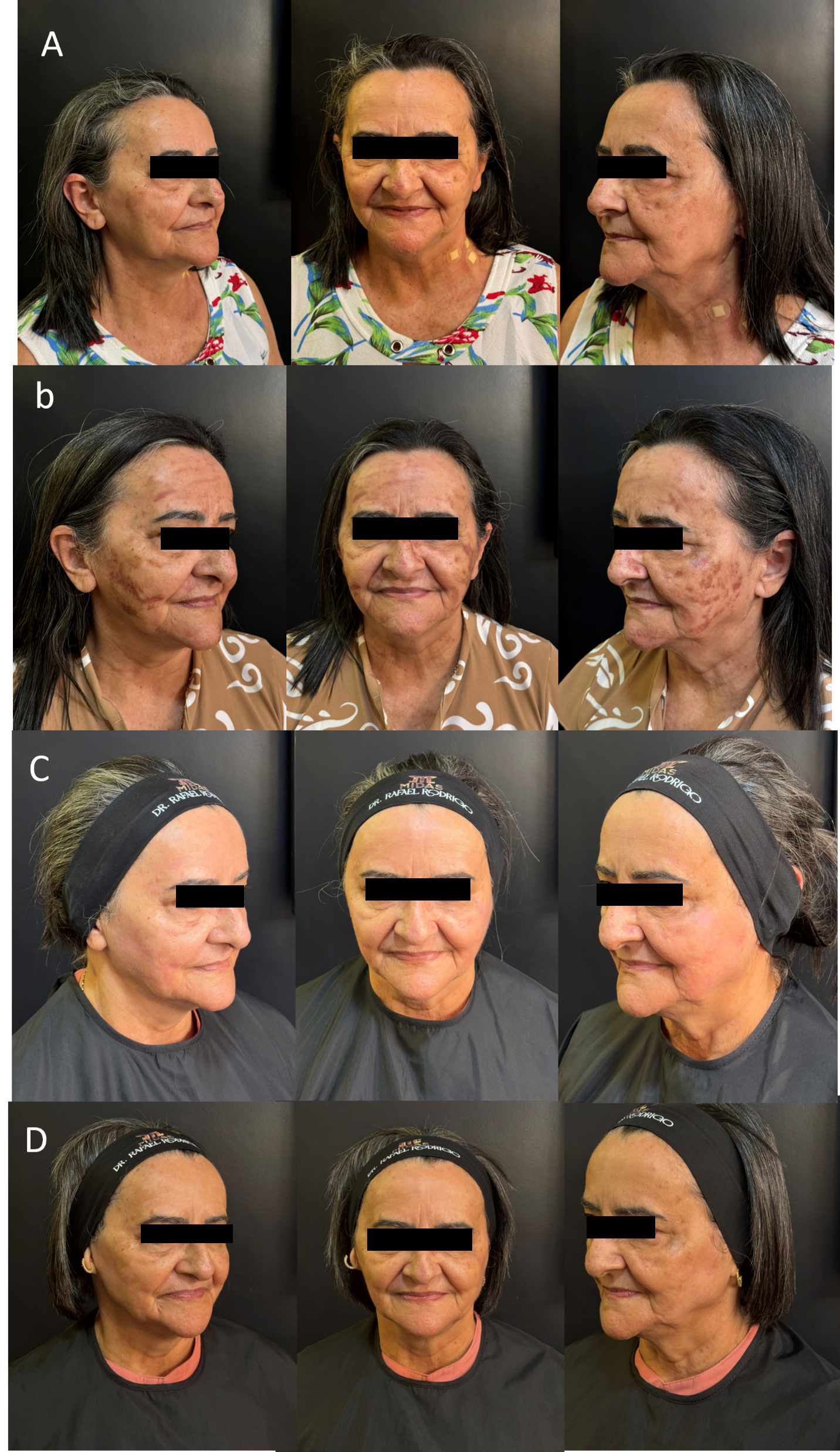

Supplement: Supplementary file 1 — Data S1: jocd70433‐sup‐0001‐Supinfo01.zip. [file JOCD-24-e70433-s001.zip › JOCD_70433_f02_Imagem revision 02.png]

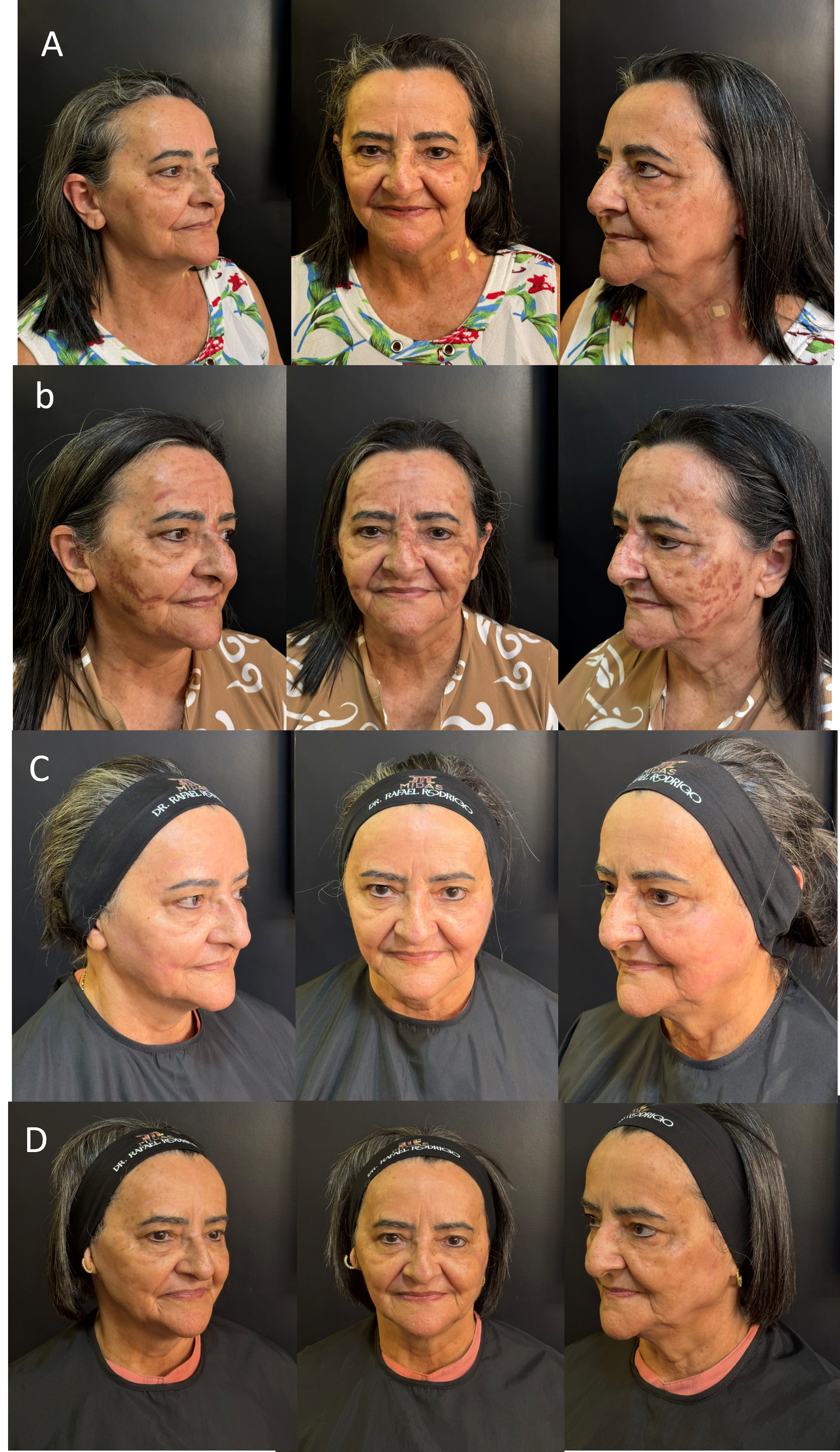

Supplement: Supplementary file 1 — Data S1: jocd70433‐sup‐0001‐Supinfo01.zip. [file JOCD-24-e70433-s001.zip › JOCD_70433_f2_Imagem2.png]
